# Temporal trends and practice variation of paediatric diagnostic tests in primary care: retrospective analysis of 14 million tests

**DOI:** 10.1136/fmch-2024-002991

**Published:** 2024-10-23

**Authors:** Elizabeth T Thomas, Diana R Withrow, Cynthia Wright Drakesmith, Peter J Gill, Rafael Perera-Salazar, Carl Heneghan

**Affiliations:** 1 Centre for Evidence-Based Medicine, Nuffield Department of Primary Care Health Sciences, University of Oxford, Oxford, UK; 2 Nuffield Department of Primary Care Health Sciences, University of Oxford, Oxford, UK; 3 Department of Pediatrics, University of Toronto, Toronto, Ontario, Canada; 4 Hospital for Sick Children, Toronto, Ontario, Canada

**Keywords:** Primary Health Care, Pediatrics

## Abstract

**Objective:**

The primary objective was to investigate temporal trends and between-practice variability of paediatric test use in primary care.

**Methods and analysis:**

This was a descriptive study of population-based data from Clinical Practice Research Datalink Aurum primary care consultation records from 1 January 2007 to 31 December 2019. Children aged 0–15 who were registered to one of the eligible 1464 general practices and had a diagnostic test code in their clinical record were included. The primary outcome measures were (1) temporal changes in test rates measured by the average annual percent change, stratified by test type, gender, age group and deprivation level and (2) practice variability in test use, measured by the coefficient of variation.

**Results:**

14 299 598 diagnostic tests were requested over 27.8 million child-years of observation for 2 542 101 children. Overall test use increased by 3.6%/year (95% CI 3.4 to 3.8%) from 399/1000 child-years to 608/1000 child-years, driven by increases in blood tests (8.0%/year, 95% CI 7.7 to 8.4), females aged 11–15 (4.0%/year, 95% CI 3.7 to 4.3), and children from the most socioeconomically deprived group (4.4% /year, 95% CI 4.1 to 4.8). Tests subject to the greatest temporal increases were faecal calprotectin, fractional exhaled nitric oxide and vitamin D. Tests classified as high-use and high-practice variability were iron studies, coeliac testing, vitamin B_12_, folate, and vitamin D.

**Conclusions:**

In this first nationwide study of paediatric test use in primary care, we observed significant temporal increases and practice variability in testing. This reflects inconsistency in practice and diagnosis rates and a scarcity of evidence-based guidance. Increased test use generates more clinical activity with significant resource implications but conversely may improve clinical outcomes. Future research should evaluate whether increased test use and variability are warranted by exploring test indications and test results and directly examine how increased test use impacts on quality of care.

**WHAT IS ALREADY KNOWN ON THIS TOPIC**

**WHAT THIS STUDY ADDS**

- In England, between 2007 and 2019, diagnostic test use increased by 4% per year, from 399 tests/1000 child-years to 608 tests/1000-child years. Test increases were driven by blood tests, especially in females 11–15 years of age, and children in the most deprived socioeconomic group. Specific tests that increased by the greatest margin include faecal calprotectin, fractional exhaled nitric oxide (FeNO) and vitamin D levels. Tests subjected to the greatest practice variation by 2019 were FeNO, hearing tests and vitamin D levels.

**HOW THIS STUDY MIGHT AFFECT RESEARCH, PRACTICE OR POLICY**

- Variability in test use highlights a lack of standardised guidance and evidence in paediatric diagnostics, which has significant implications for downstream diagnostic activity, treatment, referrals and healthcare costs.

## Introduction

 It has been reported that 70% of clinical decisions involve the use of diagnostic tests.^1^ Paediatric test use has not previously been characterised in large-scale population studies, especially in primary care, where most paediatric health contacts occur. Compared with adults, children more commonly present with undifferentiated symptoms, and non-specific reports such as abdominal pain, headaches and fatigue often have no identifiable underlying cause.^2^ While tests are one of the many diagnostic strategies available to clinicians, they must balance the risks of overinvestigation and unnecessary referrals with missing or delaying a diagnosis. It is difficult to achieve the right balance of care, and the threshold to test, treat or refer varies among clinicians,^3^ which can lead to substantial variation in the healthcare delivered to children.^4 5^

Measuring variation in testing can identify tests that are potentially overused or underused, with both overuse and underuse having potentially harmful consequences.^6^ For the patient, overuse can result in testing cascades, which can lead to unnecessary treatment and cause physical and emotional harms.^7^ For the healthcare system, overuse generates additional workload for clinicians and can lead to unnecessary referrals, healthcare contacts and health spending in an already overburdened system.^8 9^ Underuse of tests can result in missed or delayed diagnosis and treatment with potentially serious physical, emotional and financial consequences for patients, families and clinicians.

Prior studies analysing test use in adults reported that test use in primary care increased by 8.5% annually.^10 11^ We previously published a study that described temporal trends in paediatric blood tests in Oxfordshire from 2005 to 2019 and found that test use increased in outpatient (specialty and general practice) settings compared with inpatient services where test rates remained stable; however, this study was limited to blood tests and restricted to tests reported by a single laboratory.^12^ The overall aim of this study was, therefore, to quantify and analyse temporal change and variation in paediatric diagnostic tests in primary care across England. The specific objectives were to (1) quantify how paediatric diagnostic test use has changed over time (2) analyse how test rates vary by general practice and (3) examine the impact of demographic and socioeconomic factors on test rates.

## Methods

### Study design and sample

This was a retrospective population-based observational study using routinely collected data from the electronic health records of children aged 0–15 years presenting to primary care practices in the UK (99% from England, 1% from Northern Ireland) from 1 January 2007 to 31 December 2019.^13^ The UK National Health Service is a publicly funded healthcare system, where general practitioners are gatekeepers to specialist paediatric care and carry out most healthcare consultations for children.^14^ Person-years were estimated as the time from birth or registration date, until 16 years of age, death, end of the study period or transfer out of the practice.

### Data source

The Clinical Practice Research Datalink (CPRD) Aurum contains routinely collected data from primary care practices that use EMIS Web electronic patient record system software.^15^ The data encompass 19.9% of the UK population and 16.6% of UK primary care practices.^13^ To gather information on socioeconomic status, we obtained linked data for practice-level index of multiple deprivation (IMD), which is a composite measure derived from indicators for the following domains related to deprivation: income, employment, education and skills, health, housing, crime, access to services, and living environment.^16^ CPRD data are quality assured, have been shown to be representative of the national UK population due to its breadth and coverage, and have been extensively validated for use in observational research.^17^

### Included tests

For overall metrics of paediatric test use, we included all diagnostic tests, including blood tests, imaging, physiological tests, and invasive procedures such as colonoscopy. Physical examination findings, anthropometric measurements and vital signs were excluded. When analysing trends and variation in specific tests, we used a subset of tests restricted to (1) the 25 most frequently requested tests during the study period (2) tests that were reported to be frequently requested by primary care providers for children or perceived to be subjected to substantial variation in their use^18^ or (3) from other literature that focused on paediatric diagnostic test use in primary care.^12 19 20^ The resulting 35 included tests comprised approximately 80% of the total tests conducted (see online supplemental appendix table 1). Tests were grouped by type: blood tests, imaging and miscellaneous non-serum laboratory tests.

### Statistical analysis

Crude rates of test use were estimated per 1000 child-years, where a child who was registered for 1 year contributed 1 child-year of observation. Age-adjusted annual rates were standardised to the 2019 age distribution.

### Temporal variation

We used Joinpoint regression to model temporal changes in age-adjusted test rates from 2007 to 2019, which has been used in previous similar studies analysing temporal trends in test use.^10 12^ Points where significant changes in rates occurred (called joinpoints) were identified, and annual percentage changes (APC) between joinpoints were estimated. The joinpoint regression model also provided an estimate of the average APC (AAPC), a summary measure of the trend from 2007 to 2019, along with the associated p values. Age-adjusted rates, APCs and AAPCs were stratified by test type, gender, age group and IMD quintile (from 1 to 5), where 1 represented the least deprived group.

### Practice variation

Crude rates for practice variation used, as the denominator, child-years contributed by the practice in 2019, where each child contributed the full or partial years they were registered. We estimated an unadjusted coefficient of variation (CoV) by dividing the standard deviation (SD) of the unadjusted test rates by the mean. Rates of test use were by practice were adjusted using a generalised linear model with Poisson errors to account for gender (proportion of females), median age of the study population and deprivation index (IMD decile).^11^ Adjusted rates were used to calculate the adjusted CoV.

APCs and AAPCs were modelled in Joinpoint Regression software V.5.0.2.^21^ Data cleaning, management and all other analyses were performed using R V.4.3.1.^22^

### Patient and public involvement

The patient and public advisory group comprised three parents who were involved in the planning and design of this study, including consideration of which tests to include for the test-specific analyses.

## Results

### Characteristics of included participants and tests

There were 14 299 598 tests performed over 27 809 957 child-years of observation from 1 January 2007 to 31 December 2019 among 2 542 101 children, of whom 50.4% (1 282 072 of 2 542 101) were females, see table 1. 54.5% of the total tests (7 794 755 of 14 299 598 tests) were performed in females. Blood tests were the most frequently performed type of diagnostic tests (50.1%). Nearly 40% of tests (39.2%) were conducted for children aged 11–15. Overall, the median number of tests per child per year was 2 (IQR 1–3). Once stratified by age group, the median number of tests per year was 1 for all age groups under 11 and 2 (IQR 1–5) for children aged 11–15 years. Patients in more deprived practices were over-represented relative to population deciles.

**Table 1:** Characteristics of included participants and tests

	Number of tests	%
**Total**	14 299 598	100.0
**Type of test**		
Blood	7 157 882	50.1
Imaging	885 709	6.2
Miscellaneous*	6 256 007	43.7
**Gender**		
Male	6 504 010	45.5
Female	7 794 755	54.5
Indeterminate	833	0.0
**Age group**		
Under 1	1 460 767	10.2
1–5	3 186 435	22.3
6–10	4 046 845	28.3
11–15	5 605 551	39.2
**IMD quintile**		
1 (least deprived)	2 223 937	15.6
2	2 133 884	14.9
3	2 804 071	19.6
4	3 230 863	22.6
5 (most deprived)	3 906 843	27.3
	**Median tests per person per year**	**IQR**
**Overall**	2	1,3
**Gender**		
Male	1	1,3
Female	2	1,3
**Age group**		
Under 1	1	1,2
1–5	1	1,3
6–10	1	1,3
11–15	2	1,5

Miscellaneous tests include laboratory analysis of non-serum samples (eg, urine, stool) and physiological measurements.

IMDindex of multiple deprivation

### Temporal trends in overall test use

**Figure 1:**
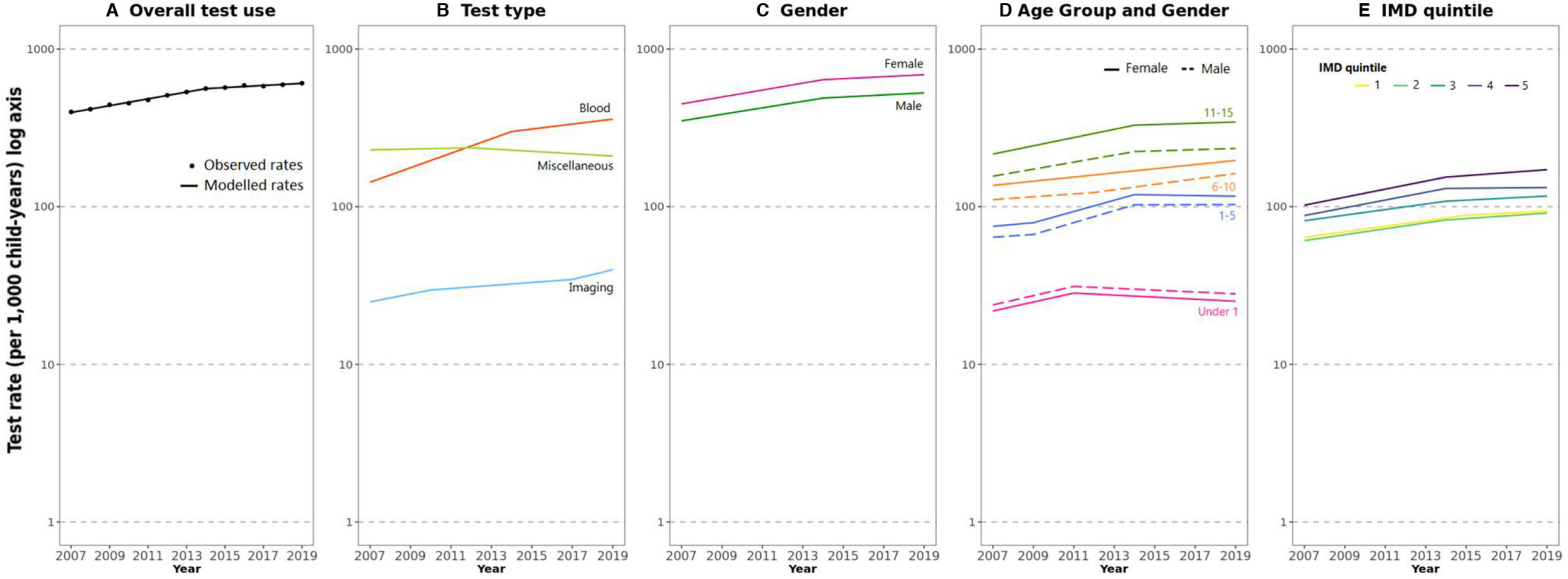
Temporal trends in paediatric test use in English primary care practices from 2007 to 2019. (A) Overall test use, (B) by test type, (C) by gender, (D) by age group and gender, and (E) by IMD quintile.

The age-adjusted rate of total test use increased from 399 tests per 1000 child years in 2007 to 608 tests per 1000 child years in 2019, an average annual percentage increase of 3.6% per year (AAPC 95% CI 3.4 to 3.8%), see figure 1A, online supplemental appendix table 1. Test rates initially increased by 5.1% per year (APC 95% CI 4.7 to 5.6%) between 2007 and 2014, then increased by 1.6% per year (APC 95% CI 0.9 to 2.1%) between 2014 and 2019. Figure 1B shows temporal changes in test use stratified by test type. The greatest increase was observed for blood tests, which increased by 8.0% per year (AAPC 95% 7.7 to 8.4%). Rates of test use by gender and age group are shown in figure 1C,D. Test rates were consistently higher in females compared with males. When stratified by age group, the rates of change were similar for both genders and age groups, except for 11–15 year-olds, where testing increased by 4.0% per year (AAPC 95% CI 3.7 to 4.3%) among females and slightly less, 3.4% per year among males (AAPC 95% CI 3.0 to 3.9%, p=0.02 for the difference between groups). Figure 1E demonstrates test use by IMD deprivation quintile. Test rates were highest in the most deprived cohort (IMD quintile 5) and increased the most, with an AAPC of 4.4% (95% CI 4.1% to 4.8%), compared with those from the lower quintiles of deprivation (online supplemental appendix table 1).

### Temporal trends in specific tests

**Figure 2:**
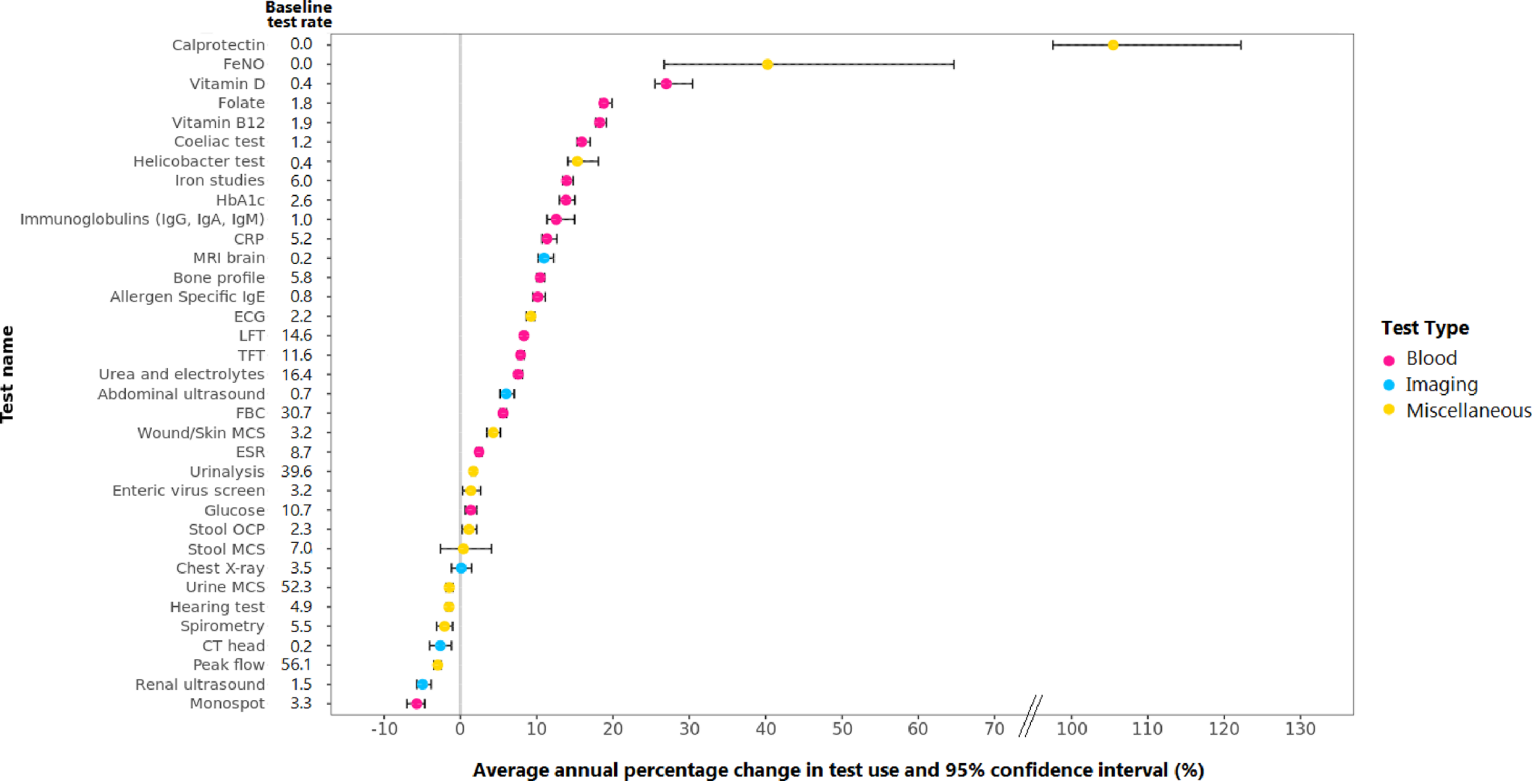
Temporal change in specific tests for children aged 0–15 years in English primary care from 2007 to 2019. FBC, full blood count; FeNO, fractional exhaled nitric oxide; LFT, liver function test; MCS, microscopy, culture, sensitivities; NOS, not otherwise specified; OCP, ova/cysts/parasites; TFT, thyroid function test; US, ultrasound.

The average APC (AAPC) for each test is presented in figure 2. The three greatest increases were for tests with zero or negligible use at the beginning of the study period. Faecal calprotectin testing was subjected to the greatest average annual change, increasing from 0 tests/1000 child-years in 2007 to 1.8 tests/1000 child-years by 2019, equivalent to 105.5% per year (AAPC 95% CI 97.5% to 122.2%). This was followed by fractional exhaled nitric oxide (FeNO) tests, which increased by 40.3% per year (AAPC 95% CI 26.7 to 64.7%) from 0 tests/1000 child-years in 2007 to 0.2 tests/1000 child-years in 2019, then vitamin D tests that increased by 27.0% per year (AAPC 95% CI 25.5 to 30.4%) from 0.4 tests/1000 child-years in 2007 to 8.5 tests/1000 child-years in 2019. The following tests increased by greater than 10% per year, listed in descending order: folate, vitamin B12, coeliac testing, helicobacter testing, iron studies, HbA1c, immunoglobulins, C reactive protein (CRP), MRI brain, bone profile and allergen-specific IgE (see online supplemental appendix table 2). Tests that decreased in use included urine microscopy/culture/sensitivities, hearing tests, spirometry, CT head, peak flow measurements, renal ultrasound and monospot testing for glandular fever. The changes were largely consistent by gender, age group (see online supplemental appendix figure 1) and deprivation quintile (see online supplemental appendix figure 2).

### Practice variation in test use

In 2019, 1464 practices contributed 2 406 042 child-years of observation (ranging from 11 to 19 553 child-years per practice). The mean rate of test use by practice (adjusted for median age, gender and deprivation level) was 609 tests per 1000 child-years (SD 41). Rates of testing varied from 0 to 2249 tests per 1000 child-years prior to adjustment, but after adjustment, the range narrowed between 424 and 732 tests per 1000 child-years (online supplemental appendix figure 3).

### Rank order of practice variability of specific tests

**Figure 3:**
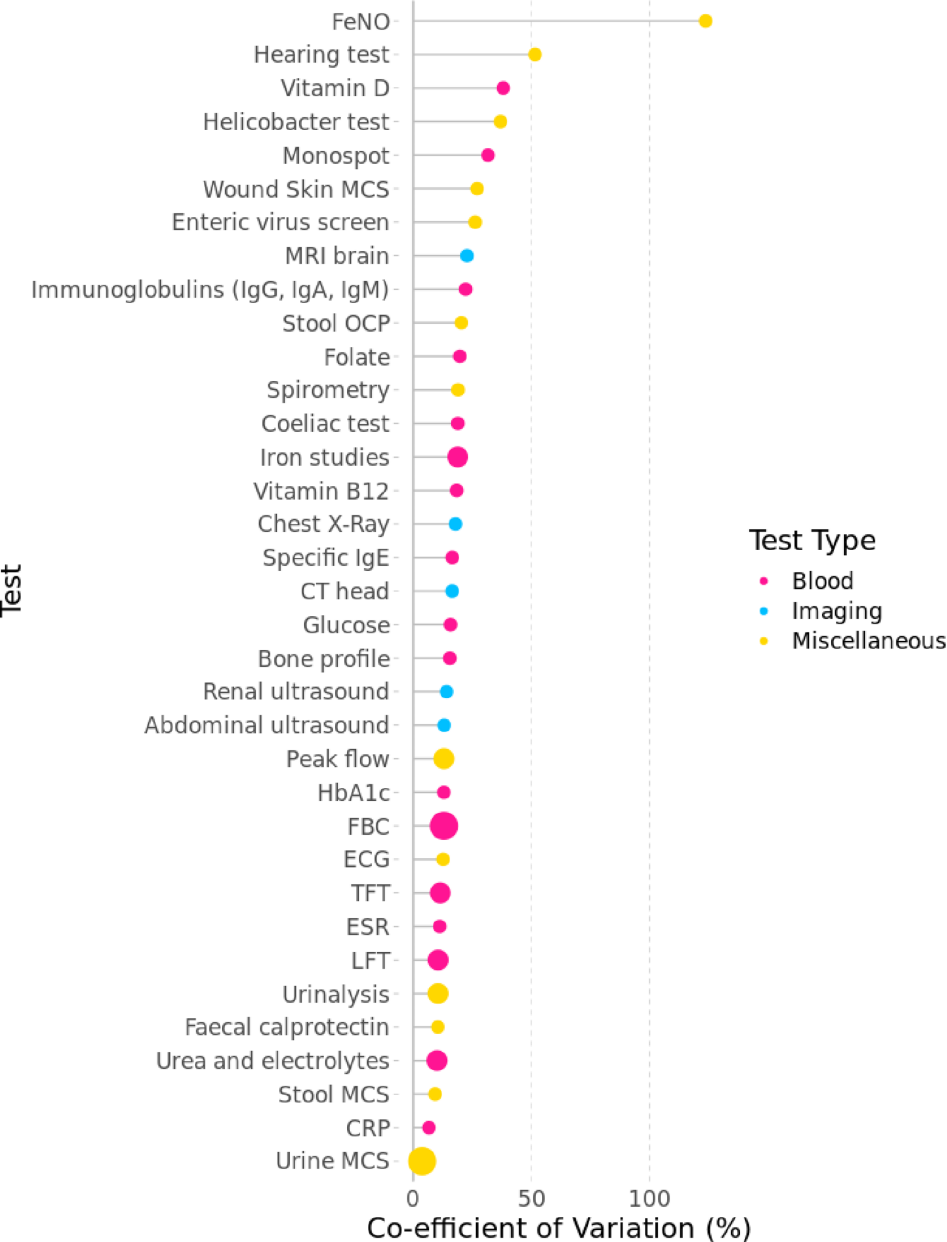
Rank order of between-practice variability of tests in 2019; adjusted for gender, age and deprivation. Tests with higher rates of use are represented by larger bubbles. CRP, C reactive protein; CXR, chest X-ray; ESR, erythrocyte sedimentation rate; FBC, full blood count; FeNO, fractional exhaled nitric oxide; LFT, liver function test; MCS, microscopy, culture, sensitivities; NOS, not otherwise specified; OCP, ova/cysts/parasites; TFT, thyroid function test.

Figure 3 shows the rank order of the tests from highest to lowest practice variability (CoV). FeNO was subjected to the greatest practice variability, with an adjusted CoV of 123.7% (95% CI 123.6 to 123.9%). This was followed by hearing tests (CoV 51.6%, 95% CI 51.4% to 51.7%) and Vitamin D tests (CoV 38.1%, 95% CI 38.0 to 38.3%). Tests with higher rates of use (represented by larger bubbles in figure 3) were, on average, subjected to lower practice variability.

**Figure 4:**
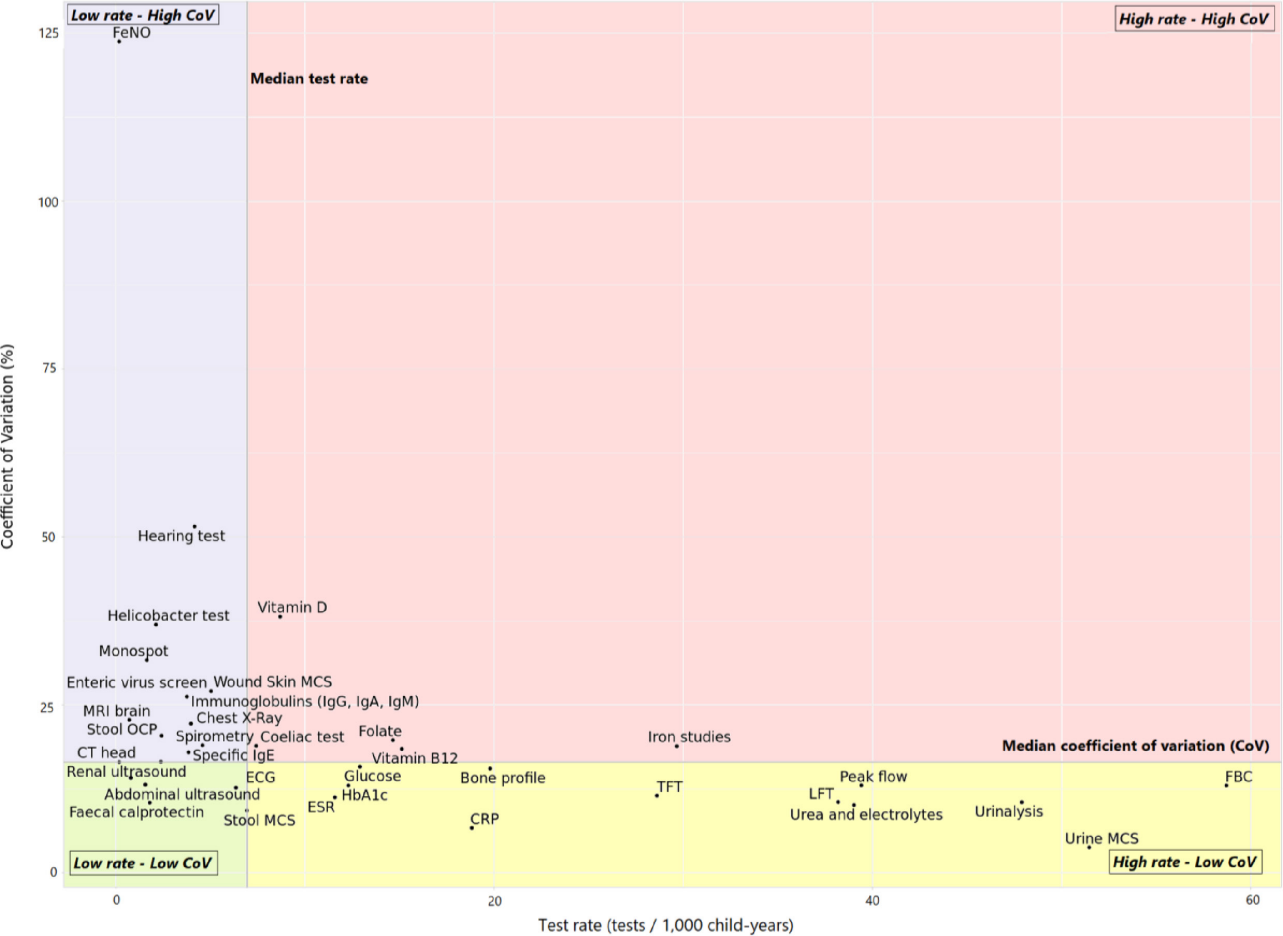
Test rate and degree of practice variability for specific tests in 2019. CRP, C reactive protein; CXR, chest X-ray; ESR, erythrocyte sedimentation rate; FBC, full blood count; FeNO, fractional exhaled nitric oxide; LFT, liver function test; MCS, microscopy, culture, sensitivities; NOS, not otherwise specified; OCP, ova/cysts/parasites; TFT, thyroid function test.

Figure 4 plots the adjusted CoV of each of the 35 tests against their test rate. The median CoV of test use was 16.5% (IQR 12.1 to 21.3%) and the median rate of test use was 6.9 tests/1000 child-years (IQR 2.4 to 19.3%). Most tests were either classified as low test rate-high variability (37%, 13 out of 35) or high test rate-low variability (37%, 13 out of 35), see online supplemental appendix table 3. The following five tests were classified as high test rate—high variability: iron studies, coeliac testing, vitamin B_12_, folate, and vitamin D.

## Discussion

To our knowledge, this study represents the first nationwide analysis of temporal trends and practice variation in paediatric test use between 2007 and 2019. We analysed 14 million tests over 27.8 million child-years of observation across nearly 1500 primary care practices and found that test use increased at a rate of 4% per year. Blood tests increased by the highest margin, and females aged 11–15 experienced the greatest increase as well as children from practices in more deprived areas. Tests with the biggest temporal increases included: faecal calprotectin, FeNO, vitamin D, folate, vitamin B_12_, coeliac and helicobacter tests. Tests subjected to the largest practice variation included FeNO, hearing tests, vitamin D, helicobacter testing and monospot testing for glandular fever. We also identified tests with high rates of test use and practice variability: iron studies, vitamin B_12_, coeliac test, folate and vitamin D.

The increasing use of both calprotectin and FeNO tests reflects the implementation of these tests in UK primary care during the study period. These two tests serve as interesting case studies: in 2019, there was low practice variability in calprotectin testing but high variability in FeNO testing. Several factors may explain this discrepancy in practice variability in 2019, including the ease with which both test samples are obtained and analysed. Additionally, it could be due to the lack of equipment, time, access and funding for FeNO testing in primary care despite evidence of their feasibility and acceptability, and national guidance recommending FeNO testing for the diagnostic workup of childhood asthma.^23^,^25^

The rise in vitamin D tests is consistent with the 42-fold increase observed for children in Minnesota from 2002 to 2017 though the odds of detecting low levels remained small, suggesting that tests were overused.^26^ Similar trends were observed in Australia and Canada.^27 28^ Although there are no specific guidelines for the paediatric population, the US Preventive Services Task Force and the American Society for Clinical Pathology guidelines for adults do not recommend screening for vitamin D deficiency.^29^ Increased vitamin D testing is, therefore, likely to represent overuse in children.

The increases observed in requests for hematinic tests (vitamin B_12_, folate, iron) were surprising and indicate growing clinician concern for nutritional deficiencies and anaemia. Similar test increases were observed for adult patients in UK primary care,^11^ which could suggest a creep of adult diagnostic practices into paediatric care.^18^ Haematinic tests, in addition to testing for coeliac disease, helicobacter pylori, vitamin D, CRP and HbA1c may also be requested for more non-specific symptoms including fatigue, musculoskeletal issues or abdominal symptoms. Further research is needed to understand the implications of the observed increases in testing and examine their appropriateness. This could be achieved by examining the test results and disease sequelae (similar to the Vitamin D studies described earlier^26 28^) or determining whether testing indications were concordant with evidence-based guidelines.

Previous research on paediatric diagnostic test use focused on specific tests and were limited to hospital settings or smaller geographic areas. A quality improvement study was conducted in paediatric intensive care units to reduce blood cultures in febrile infants, as they drive unnecessary antibiotic treatment and hospital-acquired infections.^30^ Potassium tests were also examined in a retrospective study, which revealed that increased potassium testing did not influence the need for potassium replacement in patients with status asthmaticus on continuous albuterol.^31^ Increased use of certain tests can be appropriate in the right population and setting. For example, rapid diagnostic testing for respiratory infections in the emergency setting has been shown to prevent unnecessary antibiotic prescriptions.^32^ It is, therefore, prudent to examine all test use to identify potential areas where testing may confer more benefit as well as areas where testing leads to increased harms.

Our study results concur with findings of increased blood test utilisation in Oxfordshire primary care practices; particularly for vitamin D, folate, vitamin B_12_, iron studies, coeliac testing, HbA1c, bone profile, CRP, thyroid function tests, urea and electrolytes and liver function tests.^12^ Notably, test increases were more pronounced in our nationwide study compared with Oxfordshire. This may be the result of local policies discouraging testing in primary care or the referral of children to outpatient services for testing.

We specifically looked at test utilisation in the period preceding the pandemic, however testing rates likely decreased substantially during the pandemic in accordance with decreased paediatric consultations in primary care.^33^ Assuming test rates recovered, and test rates continued at the rate of growth since 2014 (APC 1.6%), then by 2024 test rates in primary care would be 656 tests/1000 children per year. This has considerable cost implications. A vitamin D test costs approximately £10 at the Oxford University Hospitals NHS Trust Laboratories. Applying the rates of vitamin D requests to the rest of the UK population in 2019, vitamin D tests requested for children in primary care cost £2.2 million in 2019. If rates continued to increase at the AAPC of 29.6% per year, then by 2024, the rate of vitamin D testing would be 42.3 tests/1000 child-years, costing £5.4 million across UK primary care annually. While these are estimates (and assume testing rates recovered postpandemic), they provide an indication of the potential scale of the financial consequences if testing rates continued to increase. Additional harms, including physical side effects of tests from radiation, psychological distress and anxiety associated with tests, and the potential consequences that include overdiagnosis, further testing and unnecessary treatments are also important to examine and merit further research.

This study had several limitations. First, CPRD data rely on the quality of the electronic data input by clinicians using electronic health record software, which can be highly variable. Second, double counting could have occurred if both the request and the completed test were coded separately; however, this is not likely to have varied by patient demographic, practice or calendar time, therefore, the metrics of temporal trends and practice variation would nevertheless be valid. Third, there was some subjectivity in how the test names were coded and grouped. To address this, the code list of tests (and their associated panels) was developed and cross-checked with (1) existing NHS trust laboratory test lists, (2) the test codes from a previous laboratory-based study based in an NHS trust corroborated by a consultant chemical pathologist^12^ and (3) another clinician-researcher (CH). Grouped test codes (ie, ‘Immunoglobulins (IgA, IgG, IgM)’) may have obscured more subtle trends in contrast with single condition-specific test codes, which can reveal more about the diagnostic strategies for a particular disease. For example, the decline in monospot testing for glandular fever may reflect shifts in clinicians’ preferences towards alternative diagnostic strategies such as serology tests or clinical diagnosis. Finally, the findings of this nationwide study may not be generalisable to other high-income countries which have varying primary care structures and access to specialists, as well as low and middle-income countries; however, our results serve as a valuable foundation for future research that aims to compare paediatric test use across different settings.

## Conclusions

This study provides a broader picture of paediatric testing practices based on individual-level data across primary care in England. Increased testing rates can generate more clinical activity including more specialist referrals, and the potential cost implications are substantial. Future research should compare tests against clinical guideline standards and examine test results to judge whether test increases are warranted, and evaluate the downstream impact on patient outcomes and cost.

## supplementary material

10.1136/fmch-2024-002991 online supplemental file 1

## Data Availability

Data may be obtained from a third party and are not publicly available.
