# Supplementary material for: Temporal trends and practice variation of paediatric diagnostic tests in primary care: retrospective analysis of 14 million tests
Source: Fam Med Community Health. 2024 Oct 23;12(4):e002991. doi: 10.1136/fmch-2024-002991 (PMC11499842; doi:10.1136/fmch-2024-002991)
Supplement: online supplemental file 1 [file fmch-12-4-s001.pdf]

Appendices

Appendix 1 Sources of included tests

Rank	Test name	Number of tests	Source
1	Urine MCS	1391785	Top 25*
2	Urinalysis	1261359	
3	Full blood count	1249322	
4	Peak flow	1184109	
5	Urea and electrolytes	790364	
6	Liver function test	760041	
8	Thyroid function tests	560244	
9	Iron studies	489989	
10	Bone profile	375914	
11	Glucose	361960	
12	C reactive protein	349276	
13	Erythrocyte sedimentation rate	314392	
14	Vitamin B_{12}	217619	
15	Stool MCS	214476	
16	Folate	204739	
17	Vitamin D	168323	
18	HbA1c	162022	
19	Wound/Skin MCS	138975	
20	Spirometry	131770	
21	Hearing test	130290	
22	Coeliac test	120337	
23	Enteric virus screen	117247	
25	ECG	105247	
26	Chest X-Ray	104604	
29	Stool OCP	92839	
33	Immunoglobulins (IgG, IgA, IgM)	70549	Interview
34	Monospot	68121	Literature
41	Allergen Specific IgE	42642	Interview
49	Abdominal ultrasound	34195	Literature
54	Helicobacter test	30655	Interview
57	Renal ultrasound	27606	Literature
80	Calprotectin	14637	Interview
84	MRI head	12936	Literature
115	CT head	5437	Literature
197	Fractional exhaled nitric oxide	977	Interview

*Excludes unspecified tests

Appendix Table 1 Standardised rates of test use overall; stratified by test type, gender, age, and deprivation quintile; annual percentage change and average annual percentage change from 2007 to 2019

	Start	End	Rate* (per 1,000 child years) Start	End	APC (%)	95% Confidence Interval Lower Limit	Upper Limit	AAPC (%) and 95% CI
Overall	2007	2014	399.2	561.9	5.1	4.7	5.6	3.6 (3.4, 3.8)
	2014	2019	561.9	607.7	1.6	0.9	2.1	
Blood Tests	2007	2014	144,6	296.7	11.1	10.3	12	8.0 (7.7, 8.4)
	2014	2019	296.7	356.9	3.7	2.8	4.6	
Imaging	2007	2010	25.3	29.8	5.9	4.3	8.8	4.0 (3.7, 4.3)
	2010	2017	29.8	34.4	2.2	1.4	2.6	
	2017	2019	34.4	39.9	7.4	5.2	9.1	
Miscellaneous	2007	2012	229.3	236.2	0.6	-0.2	2.8	-0.7 (-1.1, -0.3)
	2012	2019	236.2	210.8	-1.7	-2.7	-1.2	
Female	2007	2014	448.6	638.7	5.2	4.7	5.7	3.6 (3.4, 3.9)
	2014	2019	638.7	690.6	1.5	0.8	2.1	
Male	2007	2014	356.3	491.2	4.9	4.4	5.4	3.5 (3.2, 3.7)
	2014	2019	491.2	528.4	1.5	0.8	2.2	
Female								
<1	2007	2011	21.5	27.3	6.8	4.4	10.3	1.2 (0.6, 1.8)
	2011	2019	27.3	25.5	-1.5	-2.5	-0.6	
1-5	2007	2009	75.6	78.8	2.7	-1.0	7.5	3.7 (3.0, 4.4)
	2009	2014	78.8	117.9	8.6	6.5	10.0	
	2014	2019	117.9	118.6	-0.5	-2.9	0.8	
6-10	2007	2019	136.3	196.4	3.1	2.3	3.9	3.1 (2.3, 3.9)
11-15	2007	2014	215.2	328.6	6.2	5.6	6.8	4.0 (3.7, 4.3)
	2014	2019	328.6	350.1	0.9	-0.1	1.9	
Male								
<1	2007	2011	23.5	30.0	7.0	4.3	11.5	1.3 (0.7, 2.1)
	2011	2019	30.0	28.7	-1.4	-2.6	-0.4	

1-5	2007	2009	64.8	66.9	2.0	-1.4	5.9	4.1 (3.4, 4.6)
	2009	2014	66.9	101.8	9.1	7.4	10.3	
	2014	2019	101.8	105.2	0.1	-2.1	1.3	
6-10	2007	2012	108.6	121.0	2.1	-0.8	3.3	3.2 (2.8, 3.7)
	2012	2019	121.0	159.1	4.0	3.3	6.7	
11-15	2007	2014	159.5	226.7	5.3	4.5	6.4	3.4 (3.0, 3.9)
	2014	2019	226.7	235.5	0.9	-0.9	2.1	
IMD 1 (least deprived quintile)	2007	2015	63.5	87.9	4.1	3.7	4.7	3.2 (3.0, 3.5)
	2015	2019	87.9	94.6	1.5	0.1	2.4	
IMD 2	2007	2014	61.4	83.5	4.4	3.9	5.6	3.4 (3.1, 3.8)
	2014	2019	83.5	92.4	2.0	0.5	2.8	
IMD 3	2007	2014	82.4	109.0	4.2	3.7	5.0	3.0 (2.8, 3.4)
	2014	2019	109.0	116.7	1.5	0.4	2.2	
IMD 4	2007	2014	88.5	130.1	5.8	5.1	6.7	3.5 (3.1, 3.9)
	2014	2019	130.1	134.4	0.3	-0.9	1.3	
IMD 5 (most deprived quintile)	2007	2014	103.4	154.4	6.0	5.4	7.0	4.4 (4.1, 4.8)
	2014	2019	154.4	169.6	2.2	0.9	3.1	

*Observed rates; Abbreviations: APC – annual percentage change; AAPC – average annual percentage change

Appendix Table 2 Average annual percentage change and test rates of 35 specific tests for children aged 0 to 15 years in primary care from 2007 to 2019

Test name	Test type	AAPC	95% CI lower limit	95% CI upper limit	2007 test rate* (tests/1,000 child years)	2019 test rate* (tests/1,000 child years)
Calprotectin	Miscellaneous	105.5	97.5	122.2	0[a]	1.8
FeNO	Miscellaneous	40.3	26.7	64.7	0[a]	0.2
Vitamin D	Blood	27.0	25.5	30.4	0.4	8.5
Folate	Blood	18.8	18.4	19.9	1.8	14.5
Vitamin B_{12}	Blood	18.3	17.7	19.1	1.9	15
Coeliac test	Blood	15.9	15.3	17.0	1.2	7.5
Helicobacter test	Miscellaneous	15.3	14.1	18.1	0.4	2.1
Iron studies	Blood	13.9	13.4	14.8	6	29.5
HbA1c	Blood	13.8	13.0	15.0	2.6	12.3
Immunoglobulins (IgG, IgA, IgM)	Blood	12.6	11.3	15.0	1	4
C reactive protein	Blood	11.3	10.7	12.6	5.2	18.8
MRI brain	Imaging	11.0	10.2	12.2	0.2	0.7
Bone profile	Blood	10.5	10.0	11.0	5.8	19.7
Allergen Specific IgE	Blood	10.1	9.5	11.1	0.8	2.37
Electrocardiogram	Miscellaneous	9.2	8.7	9.7	2.2	6.4
Liver function test	Blood	8.3	8.1	8.6	14.6	38.1
Thyroid function test	Blood	7.9	7.5	8.4	11.6	28.5
Urea and electrolytes	Blood	7.6	7.2	8.1	16.4	38.9
Abdominal ultrasound	Imaging	6.0	5.2	7.1	0.7	1.56
Full blood count	Blood	5.6	5.2	6.0	30.7	58.5
Wound/Skin MCS	Miscellaneous	4.3	3.5	5.2	3.2	5.1
Erythrocyte sedimentation rate	Blood	2.4	2.1	2.8	8.7	11.6
Urinalysis	Miscellaneous	1.7	1.4	2.0	39.6	48
Enteric virus screen	Miscellaneous	1.4	0.3	2.6	3.2	3.8
Glucose	Blood	1.4	0.6	2.1	10.7	12.8
Stool OCP	Miscellaneous	1.1	0.2	2.1	2.3	2.4
Stool MCS	Miscellaneous	0.4	-2.6	4.1	7	6.9

Chest X-ray	Imaging	0.1	-1.2	1.5	3.5	3.8
Urine MCS	Miscellaneous	-1.5	-1.9	-1.0	52.3	44.9
Hearing test	Miscellaneous	-1.5	-1.8	-1.2	4.9	4.1
Spirometry	Miscellaneous	-2.1	-3.1	-1.0	5.5	4.6
CT head	Imaging	-2.6	-4.0	-1.2	0.2	0.2
Peak flow	Miscellaneous	-3.0	-3.5	-2.5	56.1	39.4
Renal ultrasound	Imaging	-5.0	-5.7	-3.8	1.5	0.8
Monospot	Blood	-5.8	-7.0	-4.7	3.3	1.6

*Observed rates; Abbreviations: AAPC – average annual percentage change; CT – computed tomography; FeNO – fractional exhaled nitric oxide test for asthma; MCS – microscopy, culture, and sensitivities; MRI – magnetic resonance imaging; OCP – ova, cysts, and parasites

[a] There were zero tests in 2007, the first recorded calprotectin test was in 2009 and the first recorded FeNO test was in 2010

Appendix Figure 1 Temporal changes in specific tests for children aged 0 to 15 from 2007 to 2019; stratified by gender and age.

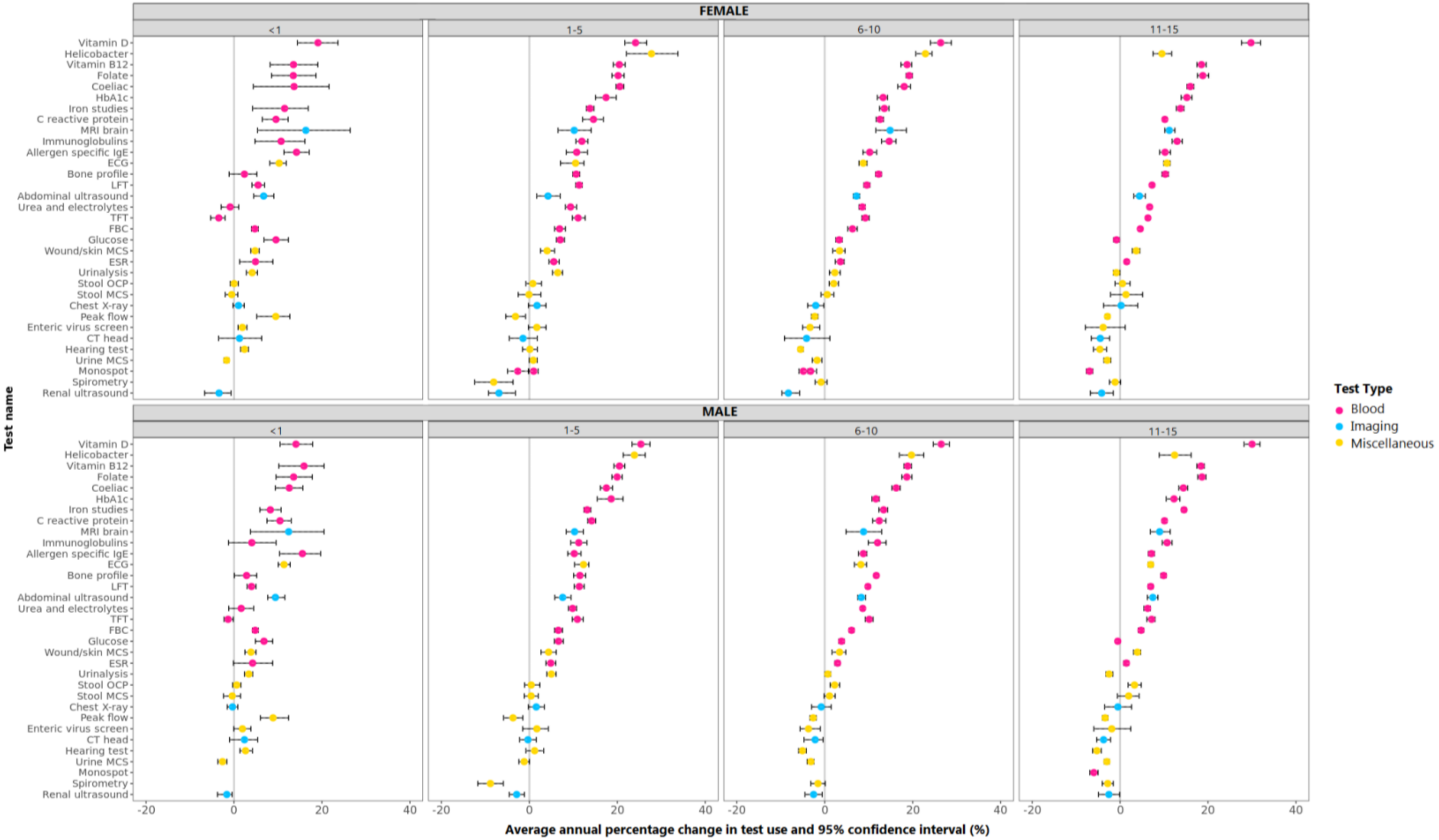

CRP – C reactive protein; ECG – Electrocardiogram; ESR – Erythrocyte sedimentation rate; FBC – Full blood count; LFT – Liver function test; MCS – Microscopy, culture, sensitivities; NOS – Not otherwise specified; OCP – Ova/cysts/parasites; TFT – Thyroid function test; US – Ultrasound. Tests were excluded if there were too few tests to perform a meaningful analysis, or the test was not technically possible to perform, leaving the outcome blank in the graph (e.g., spirometry in <1 year old).

Appendix Figure 2 Temporal changes in specific tests for children aged 0 to 15 from 2007 to 2019; stratified by Index of Multiple Deprivation Quintile.

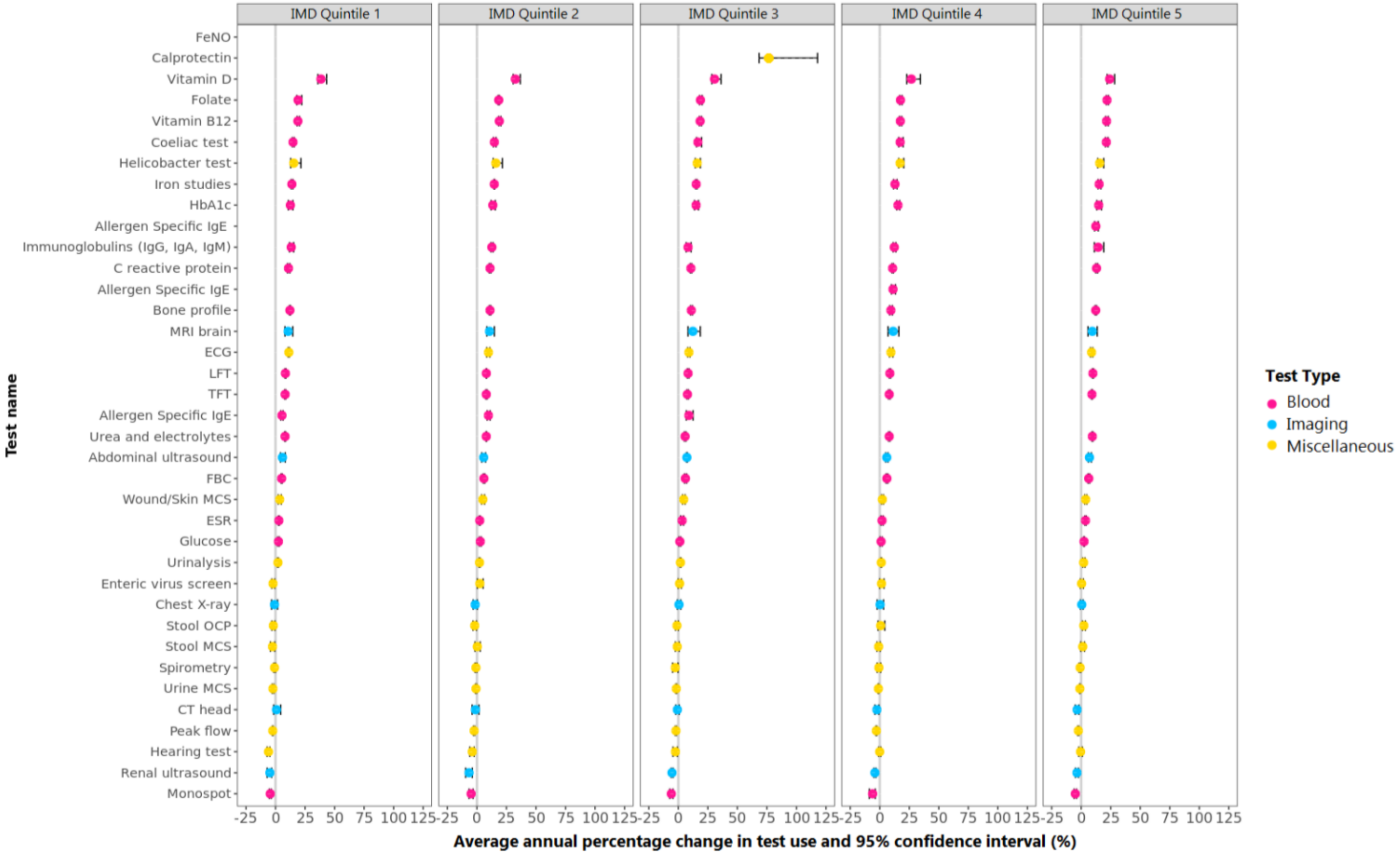

CRP – C reactive protein; ECG – Electrocardiogram; ESR – Erythrocyte sedimentation rate; FBC – Full blood count; FeNO – Fractional exhaled nitric oxide; LFT – Liver function test; MCS – Microscopy, culture, sensitivities; NOS – Not otherwise specified; OCP – Ova/cysts/parasites; TFT – Thyroid function test; US – Ultrasound.
Tests were excluded if there were too few tests to perform a meaningful analysis, leaving the outcome blank in the graph.

Appendix Figure 3 Crude and adjusted practice-specific test request rates for children aged 0 to 15 in 2019; Adjusted for gender, age, and deprivation

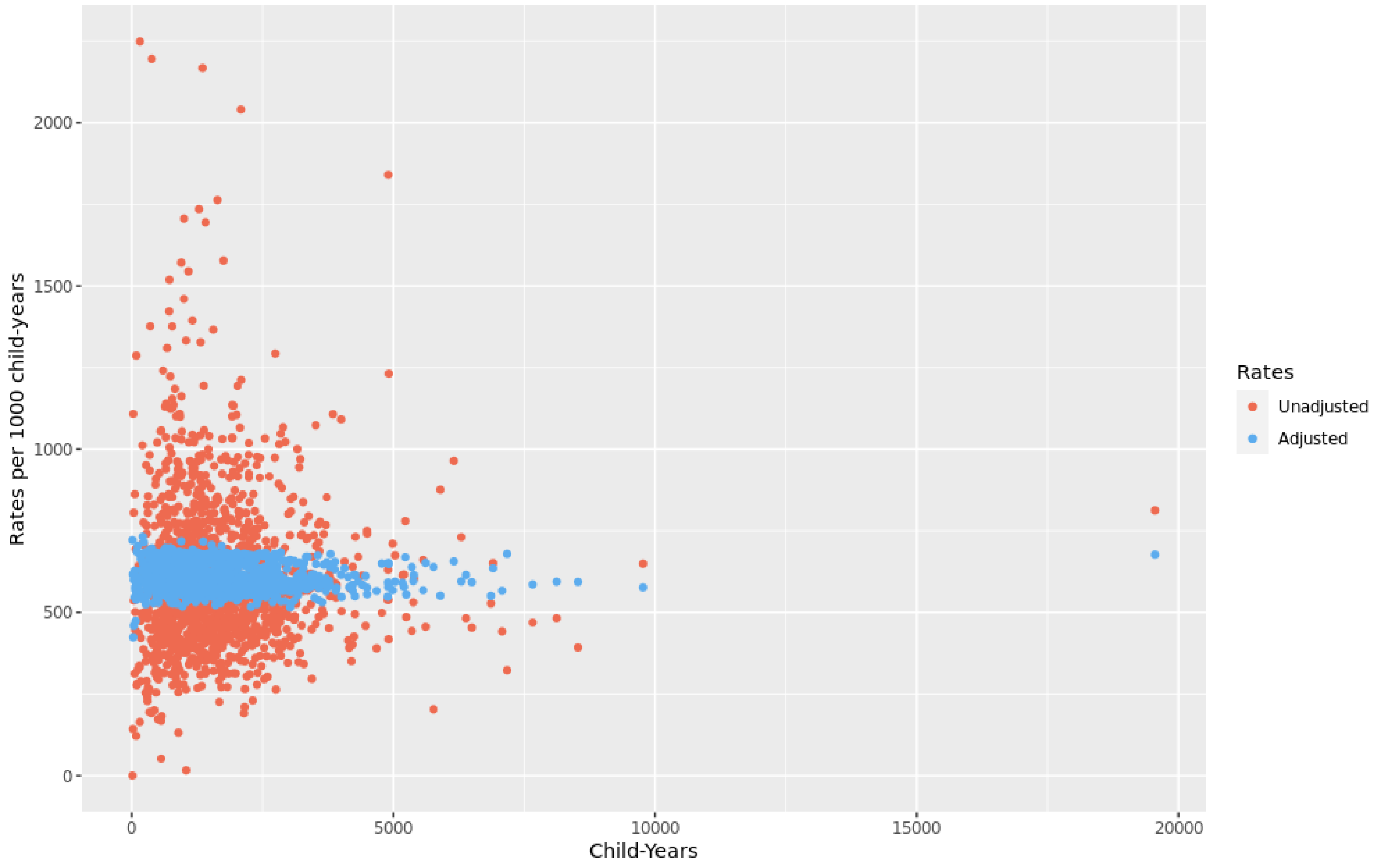

Appendix Table 3 Adjusted test rates and coefficient of variation (CoV) with corresponding 95% confidence intervals in 2019

Test name	Test type	Adjusted mean rate (tests/1,000 child-years)	Adjusted CoV (%)	95% CI lower limit (%)	95% CI upper limit (%)	Rate-Variability*
FBC	Blood	58.7	13.0	12.9	13.1	High test rate - Low CoV
Urine MCS	Miscellaneous	48.4	3.8	3.7	3.9	High test rate - Low CoV
Urinalysis	Miscellaneous	47.9	10.5	10.3	10.7	High test rate - Low CoV
Peak flow	Miscellaneous	39.4	13.0	12.8	13.3	High test rate - Low CoV
Urea and electrolytes	Blood	39.0	10.1	10.0	10.2	High test rate - Low CoV
LFT	Blood	38.2	10.5	10.4	10.7	High test rate - Low CoV
Iron studies	Blood	29.7	18.8	18.7	19.0	High test rate - High CoV
TFT	Blood	28.6	11.5	11.3	11.6	High test rate - Low CoV
Bone profile	Blood	19.8	15.5	15.4	15.7	High test rate - Low CoV
CRP	Blood	18.8	6.7	6.5	6.8	High test rate - Low CoV
Vitamin B12	Blood	15.1	18.4	18.3	18.6	High test rate - High CoV
Folate	Blood	14.6	19.7	19.6	19.9	High test rate - High CoV
Glucose	Blood	12.9	15.8	15.7	15.9	High test rate - Low CoV
HbA1c	Blood	12.3	13.0	12.9	13.1	High test rate - Low CoV
ESR	Blood	11.6	11.2	11.1	11.4	High test rate - Low CoV
Vitamin D	Blood	8.7	38.1	38.0	38.3	High test rate - High CoV
Coeliac	Blood	7.4	18.9	18.8	19.0	High test rate - High CoV
Stool MCS	Miscellaneous	6.9	9.3	9.1	9.4	High test rate - Low CoV
ECG	Miscellaneous	6.3	12.7	12.5	12.8	Low test rate - Low CoV
Wound/Skin MCS	Miscellaneous	5.0	27.1	26.9	27.2	Low test rate - High CoV
Spirometry	Miscellaneous	4.6	19.0	18.9	19.1	Low test rate - High CoV
Hearing test	Miscellaneous	4.2	51.6	51.4	51.7	Low test rate - High CoV
Immunoglobulins	Blood	4.0	22.2	22.1	22.3	Low test rate - High CoV
CXR	Imaging	3.8	17.9	17.8	18.1	Low test rate - High CoV
Enteric virus screen	Miscellaneous	3.8	26.2	26.1	26.4	Low test rate - High CoV
Stool OCP	Miscellaneous	2.4	20.4	20.3	20.5	Low test rate - High CoV
Specific IgE	Blood	2.4	16.5	16.4	16.7	Low test rate - High CoV
Helicobacter pylori	Miscellaneous	2.1	37.0	36.8	37.1	Low test rate - High CoV

Faecal calprotectin	Miscellaneous	1.8	10.4	10.3	10.6	Low test rate - Low CoV
Monospot	Blood	1.6	31.7	31.5	31.8	Low test rate - High CoV
US abdomen	Imaging	1.6	13.1	13.0	13.3	Low test rate - Low CoV
US renal	Imaging	0.8	14.1	14.0	14.3	Low test rate - Low CoV
MRI head	Imaging	0.7	22.8	22.6	22.9	Low test rate - High CoV
CT head	Imaging	0.2	16.5	16.4	16.6	Low test rate - High CoV
FeNO	Miscellaneous	0.2	123.7	123.6	123.9	Low test rate - High CoV

*High and Low in relation to test rate and variability are relative to the median test rate of 6.9 tests/1,000 child-years and median CoV of 16.5%

Abbreviations: CRP – C reactive protein; CT – computed tomography; CXR – Chest X-ray; ECG – Electrocardiogram; ESR – Erythrocyte sedimentation rate; FBC – Full blood count; FeNO – Fractional exhaled nitric oxide; LFT – Liver function test; MCS – Microscopy, culture, sensitivities; MRI – Magnetic resonance imaging; NOS – Not otherwise specified; OCP – Ova/cysts/parasites; TFT – Thyroid function test; US – Ultrasound
